# Hematologic Involvement in Systemic Lupus Erythematosus: Clinical Features and Prognostic Implications in a Hematology-Referred Cohort

**DOI:** 10.3390/jcm14207304

**Published:** 2025-10-16

**Authors:** Tuba Yuce Inel, Sadettin Uslu, Tuba Demirci Yildirim, Semih Gulle, Gercek Sen

**Affiliations:** 1 Division of Rheumatology, Izmir City Hospital, Izmir 35540, Turkey; 2 Division of Rheumatology, School of Medicine, Celal Bayar University, Manisa 45140, Turkey; 3 Division of Rheumatology, School of Medicine, Dokuz Eylül University, Izmir 35340, Turkey; semih.gulle@deu.edu.tr (S.G.); gercek.can@deu.edu.tr (G.S.)

**Keywords:** systemic lupus erythematosus, hematologic manifestations, complement, splenomegaly

## Abstract

**Background/Objectives:** Systemic lupus erythematosus (SLE) is a chronic multisystem autoimmune disease frequently complicated by hematologic abnormalities, which may reflect disease activity or treatment effects. To characterize the clinical, laboratory, and immunological features of adult SLE patients referred to hematology during routine rheumatology follow-up. **Methods:** We retrospectively analyzed 84 adult SLE patients who fulfilled the 2012 SLICC or 2019 EULAR/ACR criteria and were referred to hematology during follow-up. Clinical, laboratory, and immunological data were collected. Associations between hematologic manifestations, organ involvement, autoantibodies, and complement levels were evaluated. **Results:** The cohort included 92.6% females with a median age of 46 (IQR 36–62). Hematologic abnormalities commonly appeared within three years of disease onset. Lymphadenopathy was more frequent in patients with cutaneous vasculitis and lupus nephritis (*p* = 0.046 and *p* = 0.045). Splenomegaly was associated with serositis, anti-β2 glycoprotein I IgG, and lupus anticoagulant (LA) positivity; anti-β2GPI IgG independently predicted splenomegaly (OR 26.02, *p* = 0.006). Low C4 was associated with increased autoimmune hemolytic anemia risk (OR 5.88, *p* = 0.009), while low C3 was linked to lupus nephritis (*p* = 0.017). Antiphospholipid antibodies were significantly associated with venous thrombosis, with anti-cardiolipin IgG as an independent predictor (OR 7.43, *p* = 0.007). Stroke history, anti-histone antibodies, and higher steroid doses were associated with mortality. Remission was linked to fewer comorbidities (*p* = 0.008). **Conclusions:** Hematologic complications in SLE arise early and carry prognostic significance, with splenomegaly associated with lupus anticoagulant and anti-β2GPI IgG, and mortality linked to anti-histone antibodies.

## 1. Introduction

Systemic lupus erythematosus (SLE) is a chronic, autoimmune disease with a wide range of organ and system involvement, including hematologic abnormalities [1]. Hematologic manifestations are prevalent at the time of initial diagnosis and throughout the disease course. While mild and asymptomatic cases may not require specific therapeutic interventions, ongoing surveillance for potential cytopenias is essential for the majority of patients. The most frequently observed hematological manifestations include anemia, leukopenia, lymphopenia, thrombocytopenia, and signs such as lymphadenopathy and splenomegaly [2]. It is crucial to differentiate whether hematologic abnormalities are attributable to SLE itself or secondary to treatments administered for SLE.

Cytopenias in SLE arise from multiple, often overlapping mechanisms. Autoantibody-mediated destruction leads to premature clearance of erythrocytes, leukocytes, or platelets via splenic macrophages, contributing to autoimmune hemolytic anemia (AIHA) and immune thrombocytopenia. Complement activation, particularly through the classical pathway, promotes opsonization and phagocytosis of blood cells [3]. Bone marrow suppression due to chronic inflammation, drug toxicity, or immune-mediated infiltration can impair hematopoiesis, while abnormal T-cell activation and increased apoptosis contribute to lymphopenia. Additionally, immune complex deposition and microangiopathic processes may further exacerbate cytopenias, particularly in patients with vasculitis or antiphospholipid antibodies. Collectively, these mechanisms explain the heterogeneity of cytopenias in SLE and their association with disease activity and severity.

Leukopenia can manifest due to lymphopenia, neutropenia, or a combination of both conditions. Lymphopenia is a common white blood cell abnormality that may indicate disease activity. It often persists but is rarely severe [4]. About 12% of SLE patients have at least one episode of neutropenia during follow-up [5]. Anemia of chronic disease is the most prevalent form of anemia in SLE, accounting for approximately one-third of cases [6]. Moreover, the autoimmune process in SLE may target mature erythrocytes, causing AIHA, or affect erythroid precursors in the bone marrow, resulting in aplastic anemia. Notably, AIHA can precede the diagnosis of SLE by several years and may present as an initial manifestation [2].

Thrombocytopenia is a common hematologic manifestation of SLE, with severe forms occurring in approximately 1% of patients. It frequently coexists with other cytopenias and is often associated with the presence of antiphospholipid antibodies [7,8]. Approximately one-third of SLE patients develop lymphadenopathy, which is more frequently observed at disease onset [9], while splenomegaly tends to occur more commonly during disease flares [10].

Although hematologic involvement has been frequently reported in SLE, detailed analyses of patients referred to hematology remain limited. Our study specifically focuses on this subgroup and aims to characterize the clinical features of SLE patients co-managed in collaboration with the hematology department.

## 2. Materials and Methods

### 2.1. Patient Selection and Data Collection

**Figure 1:**
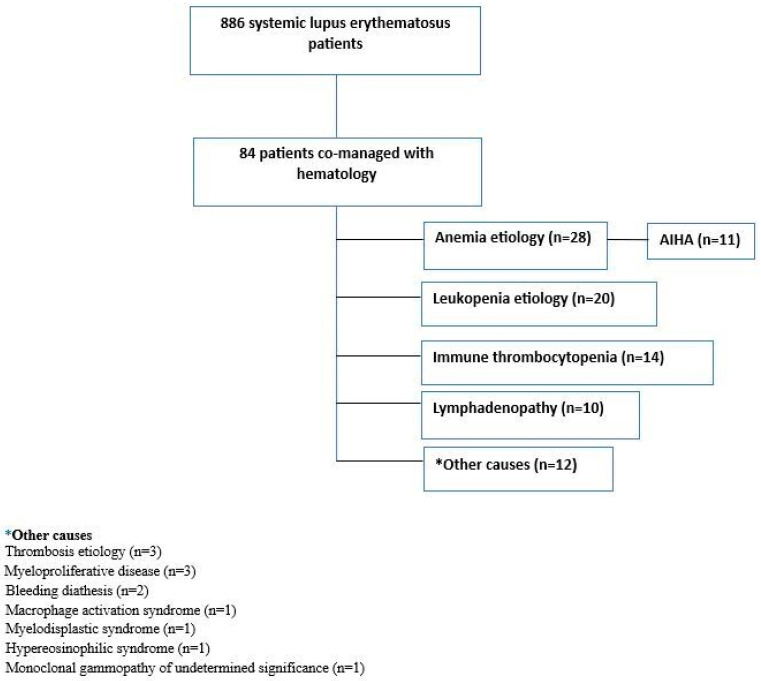
Reasons for consultation to the hematology department.

Patients were identified through the hospital database using the ICD-10 code M32 for SLE. To minimize misclassification and reduce false positives, all cases underwent detailed chart review to confirm clinical features and fulfillment of the 2012 SLICC [11] and/or 2019 EULAR/American College of Rheumatology (ACR) classification criteria [12]. This retrospective cohort included patients aged ≥18 years who met these criteria and were referred to the hematology department during follow-up. Among 886 patients identified with the ICD diagnosis code M32, the demographic, clinical, and laboratory characteristics of 84 patients referred to hematology were analyzed (Figure 1). The clinical spectrum within this cohort included hematologic abnormalities such as thrombocytopenia, hemolytic anemia, leukopenia, and hypocomplementemia. Other manifestations involved serositis and mucocutaneous, musculoskeletal, renal, neuropsychiatric, cardiovascular, and pulmonary system involvement.

Hemolytic anemia was defined by a reduction in hemoglobin levels with elevated reticulocyte counts, increased serum lactate dehydrogenase, decreased haptoglobin levels, and a positive direct Coombs test. Thrombocytopenia was defined as a platelet count of less than 100 × 10^9^/L, confirmed by peripheral blood smear examination.

### 2.2. Ethical Considerations

The study was conducted in accordance with the principles of the Declaration of Helsinki. The research protocol was approved by the Institutional Ethics Committee of Dokuz Eylul University (Approval No. 2021/18-02), and written informed consent was obtained from all participants prior to enrollment.

### 2.3. Statistical Analysis

Statistical analyses were performed using the SPSS program, version 21.0. The distribution of continuous variables was assessed using the Shapiro–Wilk test (or Kolmogorov–Smirnov test where appropriate) before selecting parametric or non-parametric statistical tests. The Chi-square test was utilized for categorical variables, while Student’s t-tests were applied to continuous variables. The Mann–Whitney test was employed for variables that did not follow a normal distribution. Variables with a *p*-value of ≤0.1 were further analyzed using univariable logistic regression. Clinically significant variables with a *p*-value of ≤0.1 were included in multivariable logistic regressions, focusing on SLE’s overall or specific hematologic manifestations. Results are reported as odds ratios (ORs) with 95% confidence intervals (CIs), with significance set at *p* ≤ 0.05.

## 3. Results

Of the patients, 92.6% (*n* = 78) were female, with a median age of 46 (IQR: 36–62). The median follow-up duration was 9 years (IQR: 5–13), and hematologic involvement was observed within the first three years of disease onset (IQR: 0–7). Patients were most frequently referred to the hematology clinic for the evaluation of anemia (*n* = 28, 33.3%), followed by leukopenia (*n* = 20, 23.8%) and immune thrombocytopenia (*n* = 14, 16.7%) (Figure 1). Among those referred for anemia, 39.2% (*n* = 11) were diagnosed with autoimmune hemolytic anemia. Coexisting autoimmune diseases were present in 41.7% of patients, the most common being Hashimoto’s thyroiditis (*n* = 13, 15.5%). A family history of rheumatic disease was reported in 11.9% of patients (*n* = 10). Most patients received corticosteroid therapy (*n* = 72, 85.7%). Renal biopsy was performed in 22.6% of the patients (*n* = 19), and only seven were compatible with lupus nephritis. The demographic and clinical characteristics of the patients are summarized in Table 1.

**Figure 2:**
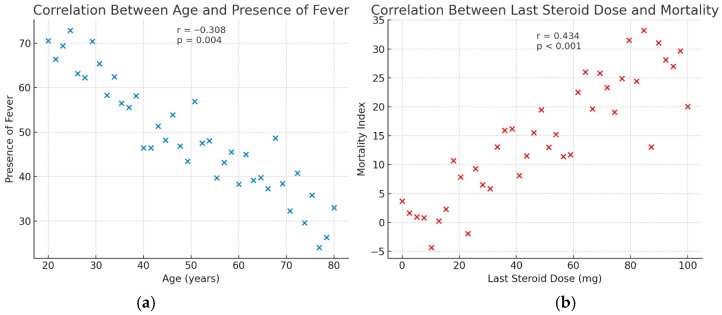
Scatter plot illustrating the correlation between (**a**) age and the presence of fever, and (**b**) the last steroid dose and mortality among patients with systemic lupus erythematosus.

With increasing age, the presentation with fever decreased (r = −0.308, *p* = 0.004) (Figure 2). Patients with serositis were significantly older than those without this manifestation (55.7 ± 15.7 vs. 46.3 ± 14.7 years, *p* = 0.032), whereas the mean age of patients with lupus nephritis was significantly lower compared to those without nephritis (33.4 ± 5.6 vs. 49.7 ± 15.3 years, *p* = 0.001). Splenomegaly was significantly associated with the presence of serositis (OR = 4.03, 95% CI: 1.23–13.11, *p* = 0.021). Furthermore, splenomegaly was associated with lupus anticoagulant and anti-β2 glycoprotein I (anti-β2GPI) IgG positivity (OR = 8.81, 95% CI: 1.70–45.57, *p* = 0.009 and OR = 5.42, 95% CI: 1.23–23.81, *p* = 0.025, respectively) (Table 2). In multivariable logistic regression analysis, anti-β2GPI IgG positivity emerged as an independent predictor of splenomegaly (OR = 26.02, 95% CI: 2.55–265.74, *p* = 0.006).

Lymphadenopathy was significantly more prevalent among patients with cutaneous vasculitis and lupus nephritis (*p* = 0.046 and *p* = 0.045, respectively). Moreover, skin-limited vasculitis was associated with anti-SSA antibody positivity (*p* = 0.020). Hypocomplementemia, specifically low C4 levels, was associated with an increased risk of autoimmune hemolytic anemia (OR = 5.88, 95% CI: 1.57–22.04, *p* = 0.009), whereas reduced C3 levels were significantly associated with the presence of lupus nephritis (*p* = 0.017). Additionally, patients exhibiting musculoskeletal involvement received a higher mean corticosteroid dose at the last visit compared to those without musculoskeletal manifestations (*p* = 0.043).

Positivity for anti-cardiolipin IgM and IgG antibodies was significantly associated with venous thrombosis (OR = 5.30, 95% CI: 1.51–18.58, *p* = 0.009 and OR = 8.00, 95% CI: 2.01–31.82, *p* = 0.003, respectively). Similarly, the presence of lupus anticoagulant and anti-β2GPI IgG antibodies was linked to an elevated risk of venous thrombosis (OR = 6.88, 95% CI: 1.68–28.24, *p* = 0.007 and OR = 9.33, 95% CI: 2.01–43.41, *p* = 0.004) (Table 2). Multivariable logistic regression analysis further identified anti-cardiolipin IgG positivity as an independent predictor of venous thrombosis (OR = 7.43, 95% CI: 1.72–32.05, *p* = 0.007).

A history of stroke was significantly associated with mortality (*p* = 0.004). Correlation analysis demonstrated a moderate positive relationship between the last steroid dose and mortality (r = 0.434, *p* < 0.001) (Figure 2). Additionally, anti-histone antibody positivity was also significantly associated with mortality (*p* = 0.019). Notably, patients who achieved remission had a substantially lower mean number of comorbidities than those who did not (0.50 ± 0.54 vs. 1.38 ± 1.43, *p* = 0.008).

## 4. Discussion

The study revealed several key findings: (1) hematologic involvement predominantly occurred within the first three years after disease onset; (2) splenomegaly was significantly associated with serositis, lupus anticoagulant, and anti-β2GPI IgG positivity; (3) lymphadenopathy was more frequent in patients with cutaneous vasculitis and lupus nephritis; and (4) mortality was associated with stroke, anti-histone antibodies, and high steroid use, while remission favored patients with fewer comorbidities.

Hematologic abnormalities are frequent in SLE and may result from diverse mechanisms, including disease activity, bone marrow failure, drug toxicity, severe infections, immune-mediated cell destruction, or neoplastic infiltration [5]. Younger age at diagnosis was associated with higher hematologic manifestations [13]. In our cohort, hematologic manifestations most commonly emerged within the first three years following disease onset.

In SLE, antiplatelet autoantibodies—predominantly of the IgG subclass—target platelet surface glycoproteins, including GpIIb/IIIa, GpIa/IIa, and GPIbIX, promoting splenic phagocytosis and subsequent thrombocytopenia despite preserved or increased megakaryocyte counts in the bone marrow [1]. The mere presence of these autoantibodies is insufficient to induce thrombocytopenia; rather, cytopenia typically develops in the context of active disease and complement activation. SLE-associated thrombocytopenia exhibits considerable clinical heterogeneity, ranging from asymptomatic cases to severe, life-threatening hemorrhagic events [14]. Thrombocytopenia frequently presented alongside other hematological manifestations [14,15,16]. Risk factors included baseline organ damage, AIHA, hypocomplementemia, anti-histone or anti-β2GPI positivity, and a positive Coombs test [16]. Moreover, patients exhibiting low levels of C3 or CH50 were found to have a higher likelihood of thrombocytopenia [17]. SLE-related thrombocytopenia was typically mild; however, its clinical significance primarily derived from its association with other severe manifestations, such as lupus nephritis and neuropsychiatric involvement, rather than from its isolated presence [18]. No significant association was observed between thrombocytopenia and other SLE-related manifestations in the present cohort. Thrombocytopenia was associated with high disease activity and independently predicted mortality [19,20,21].

Anemia affects approximately half of SLE patients and may result from various etiologies, including drug toxicity, chronic disease, iron deficiency, AIHA, or renal dysfunction [22]. Inflammation in SLE disrupts iron metabolism through hepcidin upregulation, driven by IL-6, TNF-α, IFN-γ, and IL-1, leading to reduced iron availability. Anemia of chronic disease in SLE is also associated with reduced erythropoietin activity, partly due to diminished production and the presence of anti-EPO antibodies, which may correlate with disease activity and complement consumption. Furthermore, erythropoiesis is impaired by interferon-mediated inhibition and apoptosis of erythroid progenitor cells. In AIHA, warm-type IgG antibodies, antiphospholipid antibodies, complement activation, and reduced CD59 expression collectively promote RBC destruction [1]. The present study identified AIHA in 39.2% of anemic patients. The presence of AIHA was associated with earlier disease onset, higher risk of renal involvement, seizures, serositis, and other cytopenias [23]. AIHA in SLE raised the thrombocytopenia risk in affected individuals and was linked to more severe disease, greater organ damage, and poorer long-term survival [15,24,25]. Neurological involvement has also been related with AIHA in SLE patients [26]. Low serum C4 levels in our cohort were strongly associated with an increased risk of AIHA.

Splenomegaly, although an infrequent finding in SLE, has been described as a manifestation of active disease and may arise secondary to heightened splenic immune activity, vascular congestion, or inflammatory cell infiltration. In a cohort of 940 SLE patients evaluated by computed tomography, splenomegaly was detected in 111 cases (11.8%) and persisted in approximately two-thirds, generally without major complications. Notably, 71.2% of affected patients exhibited multilineage cytopenias [27]. In our cohort, splenomegaly was significantly associated with serositis, lupus anticoagulant, and anti-β2GPI IgG positivity.

In systemic lupus erythematosus, anti-Ro/SSA antibodies cross-react with a 64 kDa neutrophil surface protein, triggering complement activation and contributing to leukopenia [28]. In the present study, cutaneous vasculitis was related to anti-SSA antibody positivity.

Anemia and lymphopenia have been identified as significant prognostic indicators in SLE, demonstrating predictive value for both disease flares and subsequent SLEDAI scores during follow-up [29]. Increased T-lymphocyte apoptosis has been observed in patients with SLE and is positively associated with disease activity [30]. Significantly, in our cohort, a lower burden of comorbidities was associated with the achievement of remission.

C3 hypocomplementemia has been linked to leukopenia, lymphopenia, and AIHA, while low C4 was primarily associated with leukopenia [31]. Prior studies in pediatric and adult SLE patients reported that low C3—but not C4—was associated with hematologic, renal, and serological abnormalities [32,33], though complement reductions were unreliable predictors of global flares [34]. Serum complement levels have also been inversely associated with lymphocyte counts independent of disease activity [35]. In our study, low C4 levels were significantly associated with an increased risk of AIHA, whereas low C3 levels were linked to the presence of lupus nephritis. Pathophysiologically, low C4 likely reflects classical pathway activation, leading to opsonization of erythrocytes and lymphocytes and contributing to AIHA and leukopenia, whereas C3 consumption is central to immune complex–mediated glomerulonephritis, explaining its association with renal involvement. These findings underscore the distinct roles of C3 and C4 in hematologic versus renal manifestations of SLE.

The primary limitations of this study include its retrospective design, single-center setting, and reliance on cumulative clinical data, which may limit the generalizability of the findings. The inability to assess antiplatelet and antileukocyte autoantibodies constitutes a limitation of our study and should be taken into account when interpreting the findings. In addition, including only patients referred to hematology introduces a potential referral bias, as these individuals may represent more severe or complex cases. Similarly, selection bias may have occurred while identifying cases from the hospital database, despite efforts to confirm diagnoses through chart review. Future prospective multicenter studies are warranted to validate these associations and further elucidate the prognostic implications of hematologic abnormalities in SLE.

Patients referred to hematology are likely to represent a more severe, complex, or refractory subset of SLE compared with unselected cohorts, which should be considered when interpreting our results. Beyond confirming previously reported associations, this study provides novel insights by demonstrating a significant relationship between splenomegaly and lupus anticoagulant and anti-β2GPI IgG positivity, and anti-histone antibodies were associated with mortality. Collaborative management between rheumatology and hematology may enhance early recognition and targeted intervention in patients with complex hematologic involvement.

## Figures and Tables

**Table 1:** Clinical and demographic characteristics of patients referred to hematology.

	*n* (%)
**Baseline characteristics**	
Gender (Female)	78 (92.6)
Age, years *	46 (36–62)
Age at diagnosis, years *	39 (24–50)
Duration at diagnosis, years *	9 (5–13)
Additional autoimmune disease	35 (41.7)
Rheumatic disease in the family	10 (11.9)
**Clinical presentation**	
Fever	10 (11.9)
Hair loss	27 (32.1)
Oral ulcer	15 (17.9)
Photosensitivity	48 (57.1)
Malar rash	39 (46.4)
Subacute cutaneous lesions	12 (14.3)
Arthritis	35 (41.7)
Arthralgia	67 (79.8)
Pleural effusion	17 (20.2)
Pericardial effusion	11 (13.1)
Dry mouth	27 (32.1)
Dry eyes	21 (25)
Raynaud	27 (32.1)
Myalgia	14 (16.7)
Neuropsychiatric involvement	9 (10.7)
Lymphadenomegaly	29 (34.5)
Splenomegaly	16 (19)
Lupus nephritis	7 (8.3)
Avascular necrosis	4 (4.8)
Thrombosis	18 (21.4)
Cutaneous vasculitis	5 (6)
**Laboratory tests**	
Anti-Ro (SS-A) positivity	23 (27.4)
Anti-La (SS-B) positivity	7 (8.3)
Anti-Sm positivity	12 (14.3)
Anti-nucleosome positivity	19 (22.6)
Anti-RNP positivity	15 (17.9)
Anti-dsDNA	48 (57.1)
Low C3	46 (54.8)
Low C4	25 (29.8)
Lupus anticoagulant	12 (14.3)
Anti-cardiolipin IgM	17 (20.2)
Anti-cardiolipin IgG	13 (15.5)
Anti-beta-2-Glycoprotein I IgM	16 (19)
Anti-beta-2-Glycoprotein I IgG	10 (11.9)
**Therapeutic interventions**	
Corticosteroid	72 (85.7)
Azathioprine	41 (48.8)
Mycophenolate mofetil	19 (22.6)
Rituximab	9 (10.7)
Cyclophosphamide	15 (17.9)
Intravenous immunoglobulin	5 (6)
Plasmapheresis	2 (2.4)

* median (IQR).

**Table 2:** Associations between serological markers and clinical outcomes (Splenomegaly and Venous Thrombosis) in patients with systemic lupus erythematosus.

*Category*	*Association*	*OR (Odds Ratio)*	*95% CI*	*p-Value*
***Splenomegaly***	Serositis	4.03	1.23–13.11	0.021
	Lupus anticoagulant	8.81	1.70–45.57	0.009
	Anti-β2GPI IgG	5.42	1.23–23.81	0.025
***Venous thrombosis***	Anti-cardiolipin IgM	5.30	1.51–18.58	0.009
	Anti-cardiolipin IgG	8.00	2.01–31.82	0.003
	Lupus anticoagulant	6.88	1.68–28.24	0.007
	Anti-β2GPI IgG	9.33	2.01–43.41	0.004

## Data Availability

The data that support the findings of this study are available from the corresponding author upon reasonable request.
